# Pain in methadone patients: Time to address undertreatment and suicide risk (ANRS-Methaville trial)

**DOI:** 10.1371/journal.pone.0176288

**Published:** 2017-05-17

**Authors:** Sandra Nordmann, Antoine Vilotitch, Caroline Lions, Laurent Michel, Marion Mora, Bruno Spire, Gwenaelle Maradan, Marc-Karim Bendiane, Alain Morel, Perrine Roux, Patrizia Carrieri, the ANRS Methaville study group

**Affiliations:** 1 Aix Marseille Université, INSERM, IRD, SESSTIM, Sciences Economiques & Sociales de la Santé & Traitement de l’Information Médicale, Marseille, France; 2 ORS PACA, Observatoire Régional de la Santé Provence-Alpes-Côte d'Azur, Marseille, France; 3 INSERM, UMR-S 669, Paris, France; 4 Université Paris-Sud and Université Paris Descartes, UMR-S 669, Paris, France; 5 Centre Pierre Nicole, Paris, France; 6 Oppelia, Paris, France; University of Toronto, CANADA

## Abstract

**Background:**

Pain in opioid-dependent patients is common but data measuring the course of pain (and its correlates) using validated scales in patients initiating methadone treatment are sparse. We aimed to assess pain and its interference in daily life, associated correlates, and undertreatment before and during methadone treatment.

**Methods:**

This is a secondary analysis using longitudinal data of a randomized trial comparing two methadone initiation models. We assessed the effect of methadone initiation and other correlates on pain intensity and interference (using the Brief Pain Inventory) at months 0, 6 and 12 using a mixed multinomial logistic regression model.

**Results:**

The study group comprised 168 patients who had data for either pain intensity or interference for at least one visit. Moderate to severe pain was reported in 12.9% of patients at M0, 5.4% at M6 and 7.3% at M12. Substantial interference with daily functioning was reported in 36.0% at M0, 14.5% at M6 and 17.1% at M12. Of the 98 visits where patients reported moderate to severe pain or substantial interference, 55.1% reported no treatment for pain relief, non-opioid analgesics were reported by 34.7%, opioid analgesics by 3.1% and both opioid and non-opioid analgesics by 7.1%. Methadone was associated with decreased pain intensity at 6 months (OR = 0.29, p = 0.04) and 12 months (OR = 0.30, p = 0.05) of follow-up and tended to be associated with substantial pain interference. Suicide risk was associated with both pain intensity and pain interference.

**Conclusions:**

Methadone in opioid-dependent patients can reduce pain. However, undertreatment of pain in methadone patients remains a major clinical concern. Patients with pain are at higher risk of suicide. Adequate screening and management of pain in this population is a priority and needs to be integrated into routine comprehensive care.

## Introduction

Methadone is a long-acting opioid agonist prescribed to treat opioid dependence and pain as a second-line treatment or in opioid rotation [1,2]. Prevalence of pain among patients on Methadone Maintenance Treatment (MMT) is estimated at 29–80% [3–6].

Among MMT patients, pain is associated with older age [4,7], a high number of comorbidities [6–11] and psychiatric disorders [6,7,9,11,12] including severe depressive symptoms [8,10,12]. Prescription and non-prescription medication use [3,4,6,9]—including prescription opioids [6,8] and benzodiazepines [4]—have also been associated with pain. Furthermore, some studies have found that pain is associated with poorer treatment outcomes such as increased use of non-prescribed opioids [9,11,13], persistence of substance use after detoxification [13] and lower retention during maintenance treatment (methadone and buprenorphine) [14].

Although one would expect methadone to reduce pain among opioid-dependent patients, given that it is a powerful analgesic, to our knowledge, no study has assessed the course of pain (and its correlates) before and during MMT using a validated scale [15].

Clinicians treating opioid-dependent patients are often faced with the complex challenge of treating pain in patients with a history of opioid-dependence [16,17]. A recent study showed that despite the high prevalence of pain among people who inject drugs, clinicians may be reluctant to prescribe opioid-based analgesics to those with a history of drug use or addiction, especially in people already enrolled in MMT [17].

We used data from the ANRS Methaville trial to 1) assess the impact of MMT initiation and other correlates on two dimensions of pain—intensity and interference—using the BPI [18] in opioid-dependent patients, 2) estimate undertreatment in this population.

## Materials and methods

### Study design

The ANRS Methaville study is a pragmatic multi-site, open-label, randomized and controlled, non-inferiority trial, which compares methadone induction in France in specialized centers (hereafter “standard care”) with methadone induction by primary care physicians. Two previous articles describe the complete protocol [19] and the primary outcomes [20]. The study was approved by the Ethics Committee for the Protection of Patients in Paris, France.

### Study group

From January 2009 to January 2010, 195 men and women were recruited in 10 sites and followed up for 12 months. Inclusion criteria were as follows: over 18 and under 70 years old, opioid-dependent in accordance with the DSM-IV criteria, and having an indication for methadone treatment (i.e. being methadone naive or not having had methadone treatment during the previous 30 days or switching from buprenorphine treatment). Exclusion criteria were as follows: having a triple (opioid, benzodiazepine and alcohol) dependence and not being reachable by phone for interview. All patients who agreed to participate in the study provided written informed consent. Subjects were identified only by number.

### Data collection

Each participant was followed up for 12 months during which he/she had 4 programmed medical visits: at enrolment and at months 3, 6 and 12 (M0, M3, M6 and M12, respectively). At each visit, a medical questionnaire and a short self-administered questionnaire were completed. A phone interview (Computer-Assisted Telephone Interview: CATI) was conducted after each visit.

#### Pain intensity, interference with daily functioning and pain treatments

We assessed pain intensity and interference (i.e. the repercussions of pain on daily functioning) using the self-administered BPI-Short Form [21] at enrolment (before methadone initiation), at M6 and at the end of the study (M12).

Pain intensity was measured using an 11-point visual analogue scale from 0 (no pain) to 10 (worst possible pain). Patients used this scale to record pain ‘‘right now,” average pain in the previous 24 hours, and their highest and lowest pain levels during the previous 24 hours. A pain intensity score was calculated when patients completed all four related questionnaire items. It equaled the average of the four different scores. We used cut-off points employed by Serlin et al., 1995 [22]. A pain rating of 0 corresponded to “no pain”, >0 and <5 to “mild pain”, and >5 to “moderate to severe pain”.

Seven additional questions on a 10-point visual scale (0: does not interfere, 10: completely interferes) evaluated pain interference in the previous 24 hours with the following seven items: general activity, mood, walking ability, normal work, relations with other people, sleep, and enjoyment of life. An interference score was obtained by averaging the scores of the interference items when at least four of the seven items were completed, as suggested by Cleeland, 1994 [18]. We used statistical cut-off points (25th and 75th interquartiles) as, to our knowledge, no standard cut-off points exit for the interference score. A rating of 0 corresponded to “no interference”, >0 and <4 to “mild interference”, and ≥ 4 to “substantial interference”.

The BPI-short Form included a question about treatments taken against pain. Analgesics were classified as follows: non-opioid analgesics, opioid analgesics and combination of non-opioid and opioid analgesics.

#### Socio-demographic characteristics

At enrolment, information on socio-demographic characteristics including gender, age, educational level, employment status, having child(ren) or not, and housing situation (renter or owner of their personal housing, living with family, living in a hospital or clinic, in a social care institution or emergency accommodation, at a friend’s home, in a hotel, and finally, being homeless) was collected. At the M12 visit, employment status and housing situation were reassessed.

#### Medication use and addictive practices

After each follow-up visit, drug use in the previous month was assessed using a section of the Opiate Treatment Index (OTI) questionnaire focusing on drug use [23]. Individuals who reported using non-prescribed opioids (i.e., illicit opioids or opioid medication obtained without a prescription), cocaine and cannabis at least once in the previous month, were defined as non-prescribed opioid, cocaine and cannabis users, respectively.

At each follow-up visit, patients who reported drug injection at least once during the previous month were defined as active injectors. Patients reporting drug injection at least once during their lifetime were defined as having a history of drug injection.

We recorded anxiolytic/hypnotic agent use as positive when at least one of the following data sources indicated their use: medical questionnaire, self-report and urine test.

Alcohol consumption was assessed at M0 and M12 during the CATI using the AUDIT questionnaire, with a cut-off point higher than 13 identifying alcohol dependence [24]. Binge drinking was defined as drinking 6 glasses or more of alcohol on one occasion at least once a month. At M0 and M6, nicotine dependence was screened for using the Fagerström test for nicotine dependence, a score ≥6 indicating high/very high dependence [25].

Daily prescribed methadone doses were recorded at M6 and M12.

During each medical visit, physicians collected data on withdrawal symptoms using the Objective Opioid Withdrawal Scale (OOWS) which comprises a list of 13 withdrawal symptoms [26].

The self-administered questionnaire at M0, M6 and M12 included 2 screening tools. The first was the Adult ADHD (Attention Deficit Hyperactivity Disorder) Self-Report Scale-Version 1.1 (ASRS-V1.1). The scoring algorithm used was the summed score obtained after adding the scores (0–4) of the first 6 items of the adult ADHD scale [27]. We defined ADHD diagnosis using 14 as a cut-off point [28]. The second tool was a 20-item self-reported inventory, the Beck hopelessness scale (BHS), where a score of 9 or more indicates suicide risk [29].

During CATI, depressive symptoms were assessed at M0, M6 and M12 using the Center for Epidemiologic Studies Depression (CES-D) scale. The validated French cut-off points of 17 for men and 23 for women was employed [30].

### Statistical analysis

We used a mixed multinomial logistic regression model to test the following factors for association with pain intensity (mild versus no pain, and moderate to severe versus no pain), and interference with daily functioning (mild versus no interference and substantial versus no interference) at M0, M6 and M12: 1) Follow-up characteristics: follow-up time (i.e. time on methadone: M6 versus M0 and M12 versus M0), methadone initiation (specialized centers versus primary care physician); 2) Socio-demographic characteristics: gender, age, educational level (< high school certificate versus ≥ high school certificate), having child(ren), employment status, stable housing (i.e. renter or owner of their personal housing versus other), receiving food assistance; 3) Addictive practices and medication use: methadone dose, anxiolytic/hypnotic agents, cocaine, non-prescribed opioids, and cannabis consumption, current drug injection, alcohol dependence, binge drinking, nicotine dependence; 4) History of drug use: switching from buprenorphine to methadone, history of drug overdose, history of drug injection, time since first regular drug use; 5) Health conditions: number of withdrawal symptoms, ADHD, current major depressive episode and suicide risk.

Variables associated with pain intensity and pain interference severity with a p-value lower than 0.20 in the univariable analysis for at least one category of the outcome were considered eligible to enter multivariable models. A stepwise procedure was used to identify the best model by removing variables one at a time based on a p-value of >0.05 for at least one of the outcome’s categories. When coefficient estimates for different categories of pain and interference were similar in the multivariable analysis, the categories were collapsed together to obtain a single estimate.

All analyses were performed using Intercooled Stata 12 (StataCorpLP, College Station, TX) software packages. The GLAMM Stata procedure was used to create a multinomial distribution.

## Results

### Description of the study group characteristics

The study group included 168 individuals with a score for pain intensity or interference at least once over the follow-up visits (M0, M6 or M12). No statistical difference was found between included (n = 168) and excluded (n = 27) patients regarding the following sociodemographic variables: age, gender, employment, housing, having children and educational level.

Among the 168 patients of the study group, 14% were female, aged 33.2 years on average (median = 32, interquartile range = [32.1–34.4]) and 31% had high school certificates. At M0, 52% were employed, 32% had unstable housing and 39% had a child(ren). Use of anxiolytic/hypnotic agents was reported by 24% of patients, cocaine by 29%, non-prescribed opioids by 72% and daily cannabis by 83%. Thirty percent had injected drugs in the previous month while 14% were alcohol dependent. Half the study group was switching from buprenorphine maintenance at trial inclusion, and 47% had a history of injection.

### Pain intensity

At M0, M6 and M12, respectively, 21.6%, 36.9% and 31.3% patients reported no pain. Moderate to severe pain was reported by 12.9% of patients at M0, 5.4% at M6 and 7.3% at M12. Average score of pain intensity varied from 2.4 (SD = 2.0) at M0 to 1.3 (SD = 1.5) at M6 and 1.5 (SD = 1.8) at M12.

In univariable analysis, mild and moderate to severe pain (versus no pain) were negatively associated with MMT at 6 and 12 months (Table 1).

**Table 1:** Factors associated with mild pain and moderate to severe pain during methadone treatment: Univariable and multivariable logistic regressions (ANRS-Methaville trial; n = 164 patients/168 included, 387 visits/403 visits).

			Univariable analysis				Multivariable analysis	
	No. of visits	No. of patients^a^	Mild pain versus no pain		Moderate to severe pain versus no pain		Pain versus no pain	
			OR [IC95%]	*p*	OR [IC95%]	*p*	OR [IC95%]	*p*
**Follow-up**								
M0	148 (38.2)	148 (90.2)	1		1		1	
M6	130 (33.6)	130 (79.3)	0.20 [0.08; 0.51]	*0*.*001*	0.10 [0.03; 0.33]	*< 0*.*001*	0.29 [0.09; 0.92]	*0*.*04*
M12	109 (28.2)	109 (66.5)	0.21 [0.08; 0.54]	*0*.*001*	0.14 [0.04; 0.48]	*0*.*002*	0.30 [0.09; 0.97]	*0*.*05*
**Medication use and addictive practices**								
**Cocaine consumption**^b^								
No	278 (78.3)	139 (86.9)	1		1			
Yes	77 (21.7)	53 (33.1)	3.35 [1.00; 11.23]	*0*.*05*	2.24 [0.52; 9.73]	*0*.*28*		
**Non-prescribed opioid consumption**^b^								
No	188 (52.2)	103 (63.2)	1		1			
Yes	172 (47.8)	119 (73.0)	3.99 [1.57; 10.15]	*0*.*004*	4.94 [1.60; 15.30]	*0*.*01*		
**History of drug use**								
**History of drug injection**								
No	266 (53.1)	83 (52.5)	1		1		1	
Yes	235 (46.9)	75 (47.5)	0.35 [0.09; 1.35]	*0*.*13*	0.28 [0.06; 1.24]	*0*.*09*	0.18 [0.04; 0.94]	*0*.*04*
**Health indicators**								
**No. of withdrawal symptoms**	0 (0–1)		1.62 [1.20; 2.19]	*0*.*002*	1.85 [1.35; 2.55]	*< 0*.*001*	1.36 [0.94; 1.95]	*0*.*10*
**Current depressive symptoms**^c^								
No	229 (68.4)	116 (75.8)	1		1			
Yes	106 (31.6)	72 (47.1)	2.44 [0.94; 6.34]	*0*.*07*	3.25 [1.01; 10.51]	*0*.*05*		
**Current suicide risk**^d^								
No	274 (72.3)	132 (81.0)	1		1		1	
Yes	105 (27.7)	69 (42.3)	4.56 [1.49; 13.91]	*0*.*01*	6.60 [1.82; 23.88]	*0*.*004*	5.90 [1.49; 23.3]	*0*.*01*
**Attention deficit hyperactivity disorder**^e^								
No	285 (74.6)	140 (85.9)	1		1			
Yes	97 (25.4)	67 (41.1)	2.86 [1.00; 8.16]	*0*.*05*	3.00 [0.85; 10.64]	*0*.*09*		

^a^ The percentages computed in this column do not add up to 100% as each refers to the proportion of individuals who had the relevant characteristics at least once during follow-up. E.g.: 73% of patients reported they consumed non-prescribed opioids at least once during follow-up.

^b^ During the previous 4 weeks

^c^ CES-D score >17 for males and >23 for females

^d^ Beck ≥ 9

^e^ ASRS score > 14

Results of the multivariable multinomial regression for mild pain and moderate to severe pain (versus no pain) were similar. For this reason, the multivariate analysis of the dependent variable pain versus no pain is presented in Table 1. This analysis showed that after adjustment for history of injection drug use and withdrawal symptoms, MMT was negatively associated with pain at 6 months (OR [95%CI] = 0.29 [0.09–0.92]; p = 0.04) and after 12 months (OR [95%CI] = 0.30 [0.09–0.97]; p = 0.05), while suicide risk was positively associated with pain (OR[95%] = 5.90 [1.49–23.3], p = 0.01).

### Interference with daily functioning

At M0, 24.5% of the study group reported no pain interference. This increased to 32.1% at M6 and 36.9% at M12. At M0, 36.0% of the patients reported substantial interference, 14.5% at M6 and 17.1% at M12. The average score of interference varied from 3.0 (SD = 2.6) at M0 to 1.7 (SD = 2.0) at M6 and 1.7 (SD = 2.0) at M12.

The results of the univariable analysis are presented in Table 2.

**Table 2:** Factors associated with mild and substantial interference during methadone treatment: Univariable and multivariable logistic regressions (ANRS-Methaville trial; n = 168 patients/168 included, 389 visits/403 visits).

			Univariable analysis				Multivariable analysis			
	No. of visits	No. of patients^a^	Mild versus no interference		Substantial versus no interference		Mild versus no interference		Substantial versus no interference	
			OR [IC95%]	*p*	OR [IC95%]	*p*	OR [IC95%]	*p*	OR [IC95%]	*p*
**Follow-up**										
M0	147 (37.8)	147 (87.5)	1		1		1		1	
M6	131 (33.7)	131 (78.0)	0.71 [0.33; 1.54]	*0*.*39*	0.21 [0.09; 0.50]	*< 0*.*001*	0.63 [0.25; 1.61]	*0*.*34*	0.36 [0.13; 1.02]	*0*.*06*
M12	111 (28.5)	111 (66.1)	0.49 [0.21; 1.10]	*0*.*08*	0.20 [0.08; 0.48]	*< 0*.*001*	0.54 [0.21; 1.42]	*0*.*21*	0.39 [0.13; 1.14]	*0*.*09*
**Socio-demographic characteristic**										
**Having child(ren)**^b^										
No	233 (60.5)	102 (61.5)	1		1					
Yes	152 (39.5)	64 (38.5)	0.48 [0.18; 1.30]	*0*.*15*	0.30 [0.11; 0.86]	*0*.*03*				
**Stable housing**^b^										
No	149 (38.4)	72 (43.1)	1				1		1	
Yes	239 (61.6)	116 (69.5)	0.34 [0.14; 0.82]	*0*.*02*	0.37 [0.14; 0.93]	*0*.*03*	0.38 [0.15; 0.98]	*0*.*05*	0.41 [0.15; 1.11]	*0*.*08*
**Medication use and addictive practices**										
**Binge drinking**^c^										
No	103 (44.4)	79 (52.7)	1		1					
Yes	129 (55.6)	95 (63.3)	2.66 [1.08; 6.57]	*0*.*03*	2.16 [0.84; 5.55]	*0*.*11*				
**Non-prescribed opioid consumption**^d^										
No	187 (51.9)	103 (61.7)	1		1					
Yes	173 (48.1)	118 (70.7)	2.05 [0.91; 4.60]	*0*.*08*	3.45 [1.45; 8.20]	*0*.*01*				
**Health indicators**										
**No. of withdrawal symptoms**	0 (0–1)		1.13 [0.90; 1.42]	*0*.*29*	1.49 [1.20; 1.85]	*< 0*.*001*	1.05 [0.80; 1.38]	*0*.*71*	1.31 [1.00; 1.70]	*0*.*05*
**Current depressive symptoms**^e^										
No	227 (68.0)	119 (76.3)	1		1					
Yes	107 (32.0)	72 (46.2)	1.53 [0.66; 3.57]	*0*.*32*	3.53 [1.45; 8.58]	*0*.*01*				
**Current suicide risk**^f^										
No	271 (71.3)	133 (80.6)	1		1		1		1	
Yes	109 (28.7)	71 (43.0)	2.37 [0.97; 5.81]	*0*.*06*	3.81 [1.50; 9.72]	*0*.*01*	2.21 [0.87; 5.67]	*0*.*10*	3.78 [1.40; 10.21]	*0*.*01*
**Attention deficit hyperactivity disorder**										
No	286 (74.7)	142 (86.1)	1		1					
Yes	97 (25.3)	66 (40.0)	1.60 [0.63; 4.08]	*0*.*32*	4.07 [1.56; 10.64]	*0*.*004*				

^a^ The percentages computed in this column do not add up to 100% as each refers to the proportion of individuals who had the characteristics at least once during follow-up. E.g.: 70.07% of patients reported they consumed non-prescribed opioids at least once during follow-up.

^b^ At M0 and M12

^c^ ≥6 glasses on the same occasion at least once a month

^d^ During the previous 4 weeks

^e^ CES-D score >17 for males and >23 for females b During the previous 6 months

^f^ Beck ≥ 9

After adjusting for potential confounding factors (withdrawal symptoms and stable housing), being at suicide risk (OR [95%CI] = 3.78 [1.40–10.21]; p = 0.01) was positively associated with substantial interference (Table 2). MMT at M6 and M12 tended to be associated with a decrease in substantial interference (respectively OR [95%CI] = 0.36 [0.13–1.02]; p = 0.06 and OR [95%CI] = 0.39 [0.13–1.14]; p = 0.09).

### Pain treatment

At M0, among the 55 patients reporting moderate to severe pain with substantial interference, 30 declared receiving no analgesic treatment, 13 opioid analgesics and 12 non-opioid analgesics. At M6, 20 patients reported moderate to severe pain with substantial interference, among them 11 declared receiving no analgesic treatment and 9 non-opioid analgesics. At M12, 23 patients reported moderate to severe pain with substantial interference, among them 11 declared receiving no analgesic treatment and 12 non-opioid analgesics.

## Discussion

There are 3 main results of this study: 1) pain decreases with methadone treatment; 2) pain is considerably undertreated in MMT patients; 3) suicide risk is a major correlate of pain intensity and pain interference with daily functioning.

At M0, 30 out of 55 patients reporting moderate to severe pain with substantial interference declared receiving no pain treatment, 11 out of 20 at M6 and 11 out of 23 at M12. This suggests that pain was inadequately treated, which is in line with other studies where pain of MMT patients was undertreated [31,32]. One explanation could be that clinicians may have deny additional analgesics to individuals already on methadone treatment [17]. Undertreatment of pain may have a negative effect on opioid dependence outcomes as many opioid maintenance treatment patients report frustration which encourages them to use illicit opioids for pain relief [17,33,34].

As shown by our results, methadone can reduce pain among opioid-dependent patients. However, its standard once-a-day administration for the treatment of opioid addiction often fails to provide sustained pain relief [35]. According to Blinderman et al., in order to treat pain, patients on MMT should receive additional methadone doses three to four times daily or every 6 to 8 hours [36]. A root cause of undertreated pain in MMT patients may be the tendency for clinicians to dichotomize MMT as either a treatment for pain or for addiction, rather than recognizing the comorbid nature of these two dimensions in individuals with a history of substance use [37]. Evidence-based guidelines have already been drawn up for pain management in opioid-dependent and MMT patients [38–40]. These guidelines should be put into clinical practice.

Suicide risk was significantly and independently associated with both pain intensity and interference in our study. Many factors may lead to suicide in pain patients. First, many experience concomitant depression [41]. Moreover, in such patients, depression may be misdiagnosed due to shared somatic symptoms between pain and depression [42]. This could explain the association between depression and pain intensity and interference found in univariable analyses in our study (this was not confirmed in multivariable analysis, probably due to collinearity with suicide risk). Second, pain catastrophizing (exaggerated, negative focus on pain) can contribute to depression, pain intensity, disability [43] and mental defeat [44]. In fact, magnitude of depression and pain catastrophizing can predict the occurrence and degree of suicidal ideation [45]. Third, sleep disorders could mediate the relationship between pain and suicide attempt/ideation. Although not observed in the present study, the association between sleep disorders and pain and suicide risk was highlighted in a previous study using Methaville trial data [46]. Fourth, physical pain could lead to suicide attempt/ideation through its interaction with social pain (hopelessness, loss of work and changing family role, as well as isolation due to pain), as both types of pain may share some underlying neurological mechanisms [47]. Finally, through indicated and appropriate prescription of opioids, vulnerable patients may receive access to potentially lethal medications (i.e., opioids) [42].

Whether methadone can reduce suicide risk through pain relief is still unknown. A previous study using data from the Methaville trial showed that methadone and suicide risk were associated in univariable analysis but no longer associated after multiple adjustment. This is probably because of collinearity between suicide risk and the number of health problems, which are significantly reduced during methadone treatment [48]. Our results suggest the need for routine assessment of pain and suicide risk by physicians, particularly in opioid-dependent patients. This is particularly relevant for patients classified as polydrug users and those with harmful alcohol consumption, considering the relatively high frequency of suicide and suicide attempts–including voluntary overdoses–in people who use drugs, whether or not they are on methadone treatment.

ADHD was associated in univariable analyses with pain intensity and interference. A recent study showed that patients with ADHD are more likely to experience pain, and that while common mental disorder influences this association, it does not fully explain the reason for this [49].

The challenge of treatment decisions by physicians regarding pain in opioid-dependent populations is compounded by the conflicting literature on the association between pain and addiction. Although some previous surveys demonstrated a link between pain and drug use behaviors [7,10,13,50–52], the present study, like others [3,8,53,54], did not find that patients with pain have a higher risk of substance use. However, univariable analyses in our study showed that pain intensity and interference were associated with non-prescribed opioid use. Whether or not treating pain in opioid-dependent patients leads to a reduction in substance use remains an important issue for future research.

Our study has strengths and limitations. The main strengths are that as a pragmatic trial, the study population is representative of people seeking care for opioid dependence in France. Second, the assessment of pain is based on a validated scale, the BPI-short Form. In terms of limitations, the study is based on self-reports to measure medication and drug use. However, the reliability of self-reports in people who use drugs has already been widely demonstrated [55].

## Conclusion

Methadone in opioid-dependent patients can reduce pain. However, undertreatment of pain in MMT patients remains a major clinical concern. Patients presenting pain and reporting interference with their daily life are at higher risk of suicide. Adequate screening and management of pain in this population is a priority and needs to be incorporated in routine comprehensive care.

## References

[1] Santiago-Palma J, Khojainova N, Kornick C, Fischberg DJ, Primavera LH, Payne R, et al Intravenous methadone in the management of chronic cancer pain: safe and effective starting doses when substituting methadone for fentanyl. Cancer. 2001;92: 1919–1925. 11745266 10.1002/1097-0142(20011001)92:7<1919::aid-cncr1710>3.0.co;2-g

[2] Shaiova L, Berger A, Blinderman CD, Bruera E, Davis MP, Derby S, et al Consensus guideline on parenteral methadone use in pain and palliative care. Palliat Support Care. 2008;6: 165–176. 10.1017/S1478951508000254 18501052

[3] Dhingra L, Perlman DC, Masson C, Chen J, McKnight C, Jordan AE, et al Longitudinal analysis of pain and illicit drug use behaviors in outpatients on methadone maintenance. Drug Alcohol Depend. 2015;149: 285–289. 10.1016/j.drugalcdep.2015.02.007 25735466 PMC4391061

[4] Dunn KE, Brooner RK, Clark MR. Severity and interference of chronic pain in methadone-maintained outpatients. Pain Med Malden Mass. 2014;15: 1540–1548. 10.1111/pme.12430 24703517

[5] Eyler ECH. Chronic and acute pain and pain management for patients in methadone maintenance treatment. Am J Addict Am Acad Psychiatr Alcohol Addict. 2013;22: 75–83. 10.1111/j.1521-0391.2013.00308.x 23398230

[6] Voon P, Hayashi K, Milloy M-J, Nguyen P, Wood E, Montaner J, et al Pain Among High-Risk Patients on Methadone Maintenance Treatment. J Pain Off J Am Pain Soc. 2015;16: 887–894. 10.1016/j.jpain.2015.06.003 26101814 PMC4556532

[7] Rosenblum A, Joseph H, Fong C, Kipnis S, Cleland C, Portenoy RK. Prevalence and characteristics of chronic pain among chemically dependent patients in methadone maintenance and residential treatment facilities. JAMA. 2003;289: 2370–2378. 10.1001/jama.289.18.2370 12746360

[8] Dhingra L, Masson C, Perlman DC, Seewald RM, Katz J, McKnight C, et al Epidemiology of pain among outpatients in methadone maintenance treatment programs. Drug Alcohol Depend. 2013;128: 161–165. 10.1016/j.drugalcdep.2012.08.003 22951068 PMC3546120

[9] Jamison RN, Kauffman J, Katz NP. Characteristics of methadone maintenance patients with chronic pain. J Pain Symptom Manage. 2000;19: 53–62. 10687327 10.1016/s0885-3924(99)00144-x

[10] Potter JS, Shiffman SJ, Weiss RD. Chronic pain severity in opioid-dependent patients. Am J Drug Alcohol Abuse. 2008;34: 101–107. 10.1080/00952990701523706 18161648 PMC3579615

[11] Trafton JA, Oliva EM, Horst DA, Minkel JD, Humphreys K. Treatment needs associated with pain in substance use disorder patients: implications for concurrent treatment. Drug Alcohol Depend. 2004;73: 23–31. 14687956 10.1016/j.drugalcdep.2003.08.007

[12] Barry DT, Beitel M, Garnet B, Joshi D, Rosenblum A, Schottenfeld RS. Relations among psychopathology, substance use, and physical pain experiences in methadone-maintained patients. J Clin Psychiatry. 2009;70: 1213–1218. 10.4088/JCP.08m04367 19607760 PMC2760669

[13] Larson MJ, Paasche-Orlow M, Cheng DM, Lloyd-Travaglini C, Saitz R, Samet JH. Persistent pain is associated with substance use after detoxification: a prospective cohort analysis. Addict Abingdon Engl. 2007;102: 752–760. 10.1111/j.1360-0443.2007.01759.x 17506152

[14] Bounes V, Palmaro A, Lapeyre-Mestre M, Roussin A. Long-term consequences of acute pain for patients under methadone or buprenorphine maintenance treatment. Pain Physician. 2013;16: E739–747. 24284855

[15] Cleeland CS, Nakamura Y, Mendoza TR, Edwards KR, Douglas J, Serlin RC. Dimensions of the impact of cancer pain in a four country sample: new information from multidimensional scaling. Pain. 1996;67: 267–273. 8951920 10.1016/0304-3959(96)03131-4

[16] Breitbart W, Rosenfeld B, Passik S, Kaim M, Funesti-Esch J, Stein K. A comparison of pain report and adequacy of analgesic therapy in ambulatory AIDS patients with and without a history of substance abuse. Pain. 1997;72: 235–243. 9272808 10.1016/s0304-3959(97)00039-0

[17] Voon P, Callon C, Nguyen P, Dobrer S, Montaner JSG, Wood E, et al Denial of prescription analgesia among people who inject drugs in a Canadian setting. Drug Alcohol Rev. 2015;34: 221–228. 10.1111/dar.12226 25521168 PMC4657858

[18] Cleeland CS, Ryan KM. Pain assessment: global use of the Brief Pain Inventory. Ann Acad Med Singapore. 1994;23: 129–138. 8080219

[19] Roux P, Michel L, Cohen J, Mora M, Morel A, Aubertin J-F, et al Methadone induction in primary care (ANRS-Methaville): a phase III randomized intervention trial. BMC Public Health. 2012;12: 488 10.1186/1471-2458-12-488 22741944 PMC3528472

[20] Carrieri PM, Michel L, Lions C, Cohen J, Vray M, Mora M, et al Methadone induction in primary care for opioid dependence: a pragmatic randomized trial (ANRS Methaville). PloS One. 2014;9: e112328 10.1371/journal.pone.0112328 25393311 PMC4231094

[21] Poundja J, Fikretoglu D, Guay S, Brunet A. Validation of the French version of the brief pain inventory in Canadian veterans suffering from traumatic stress. J Pain Symptom Manage. 2007;33: 720–726. 10.1016/j.jpainsymman.2006.09.031 17531912

[22] Serlin RC, Mendoza TR, Nakamura Y, Edwards KR, Cleeland CS. When is cancer pain mild, moderate or severe? Grading pain severity by its interference with function. Pain. 1995;61: 277–284. 7659438 10.1016/0304-3959(94)00178-H

[23] Darke S, Ward J, Hall W, Heather N, Wodak A. The Opiate Treatment Index (OTI) researchers’ manual. 1991; 1–66.

[24] Gache P, Michaud P, Landry U, Accietto C, Arfaoui S, Wenger O, et al The Alcohol Use Disorders Identification Test (AUDIT) as a screening tool for excessive drinking in primary care: reliability and validity of a French version. Alcohol Clin Exp Res. 2005;29: 2001–2007. 16340457 10.1097/01.alc.0000187034.58955.64

[25] Heatherton TF, Kozlowski LT, Frecker RC, Fagerström KO. The Fagerström Test for Nicotine Dependence: a revision of the Fagerström Tolerance Questionnaire. Br J Addict. 1991;86: 1119–1127. 1932883 10.1111/j.1360-0443.1991.tb01879.x

[26] Handelsman L, Cochrane KJ, Aronson MJ, Ness R, Rubinstein KJ, Kanof PD. Two new rating scales for opiate withdrawal. Am J Drug Alcohol Abuse. 1987;13: 293–308. 10.3109/00952998709001515 3687892

[27] van de Glind G, van den Brink W, Koeter MWJ, Carpentier P-J, van Emmerik-van Oortmerssen K, Kaye S, et al Validity of the Adult ADHD Self-Report Scale (ASRS) as a screener for adult ADHD in treatment seeking substance use disorder patients. Drug Alcohol Depend. 2013;132: 587–596. 10.1016/j.drugalcdep.2013.04.010 23660242 PMC4083506

[28] Daigre C, Roncero C, Rodríguez-Cintas L, Ortega L, Lligoña A, Fuentes S, et al Adult ADHD screening in alcohol-dependent patients using the Wender-Utah Rating Scale and the adult ADHD Self-Report Scale. J Atten Disord. 2015;19: 328–334. 10.1177/1087054714529819 24743975

[29] Beck AT, Weishaar ME. Suicide risk assessment and prediction. Crisis. 1990;11: 22–30. 2076612

[30] Führer R, Rouillon F. La version française de l’échelle CES-D (Center for Epidemiologic Studies-Depression Scale). Description et traduction de l’échelle d’auto-évaluation. Psychiatrie et Psychobiologie. 1989: 163–166.

[31] Hines S, Theodorou S, Williamson A, Fong D, Curry K. Management of acute pain in methadone maintenance therapy in-patients. Drug Alcohol Rev. 2008;27: 519–523. 10.1080/09595230802245519 18696299

[32] Rowley D, McLean S, O’Gorman A, Ryan K, McQuillan R. Review of cancer pain management in patients receiving maintenance methadone therapy. Am J Hosp Palliat Care. 2011;28: 183–187. 10.1177/1049909110380897 20826493

[33] Karasz A, Zallman L, Berg K, Gourevitch M, Selwyn P, Arnsten JH, et al The experience of chronic severe pain in patients undergoing methadone maintenance treatment. J Pain Symptom Manage. 2004;28: 517–525. 10.1016/j.jpainsymman.2004.02.025 15504628

[34] St Marie B. Health care experiences when pain and substance use disorder coexist: “just because i’m an addict doesn’t mean i don’t have pain.” Pain Med Malden Mass. 2014;15: 2075–2086. 10.1111/pme.12493 25041442 PMC4300296

[35] Fishman SM, Wilsey B, Mahajan G, Molina P. Methadone reincarnated: novel clinical applications with related concerns. Pain Med Malden Mass. 2002;3: 339–348. 10.1046/j.1526-4637.2002.02047.x 15099239

[36] Blinderman CD, Sekine R, Zhang B, Nillson M, Shaiova L. Methadone as an analgesic for patients with chronic pain in methadone maintenance treatment programs (MMTPs). J Opioid Manag. 2009;5: 107–114. 19507807 10.5055/jom.2009.0012

[37] Keane H. Categorising methadone: Addiction and analgesia. Int J Drug Policy. 2013;24: e18–24. 10.1016/j.drugpo.2013.05.007 23768774

[38] Center for Substance Abuse Treatment. Clinical Guidelines for the Use of Buprenorphine in the Treatment of Opioid Addiction Treatment Improvement Protocol (TIP) Series 40. DHHS Publication No. (SMA) 04–3939. Rockville, MD: Substance Abuse and Mental Health Services Administration 2004. 22514846

[39] Center for Substance Abuse Treatment. Medication-assisted treatment for opioid addiction in opioid treatment programs Rockville, MD: Substance Abuse and Mental Health Services Administration Treatment Improvement Protocol (TIP) Series 43. HHS Publication No. (SMA) 05–4048. 2005. 22514849

[40] Center for Substance Abuse Treatment. Managing Chronic Pain in Adults With or in Recovery From Substance Use Disorders Treatment Improvement Protocol (TIP) Series 54. HHS Publication No. (SMA) 12–4671. Rockville, MD: Substance Abuse and Mental Health Services Administration 2011. 22514862

[41] Miller LR, Cano A. Comorbid chronic pain and depression: who is at risk? J Pain Off J Am Pain Soc. 2009;10: 619–627. 10.1016/j.jpain.2008.12.007 19398383

[42] Cheatle MD. Depression, chronic pain, and suicide by overdose: on the edge. Pain Med Malden Mass. 2011;12 Suppl 2: S43–48. 10.1111/j.1526-4637.2011.01131.x 21668756 PMC3125689

[43] Turner JA, Jensen MP, Warms CA, Cardenas DD. Catastrophizing is associated with pain intensity, psychological distress, and pain-related disability among individuals with chronic pain after spinal cord injury. Pain. 2002;98: 127–134. 12098624 10.1016/s0304-3959(02)00045-3

[44] Tang NKY, Beckwith P, Ashworth P. Mental Defeat Is Associated With Suicide Intent in Patients With Chronic Pain. Clin J Pain. 2016;32: 411–419. 10.1097/AJP.0000000000000276 26201013

[45] Edwards RR, Smith MT, Kudel I, Haythornthwaite J. Pain-related catastrophizing as a risk factor for suicidal ideation in chronic pain. Pain. 2006;126: 272–279. 10.1016/j.pain.2006.07.004 16926068

[46] Nordmann S, Lions C, Vilotitch A, Michel L, Mora M, Spire B, et al A prospective, longitudinal study of sleep disturbance and comorbidity in opiate dependence (the ANRS Methaville study). Psychopharmacology (Berl). 2016;233: 1203–1213. 10.1007/s00213-016-4202-4 26753792

[47] Eisenberger NI. The neural bases of social pain: evidence for shared representations with physical pain. Psychosom Med. 2012;74: 126–135. 10.1097/PSY.0b013e3182464dd1 22286852 PMC3273616

[48] Michel L, Lions C, Maradan G, Mora M, Marcellin F, Morel A, et al Suicidal risk among patients enrolled in methadone maintenance treatment: HCV status and implications for suicide prevention (ANRS Methaville). Compr Psychiatry. 2015;62: 123–131. 26343476 10.1016/j.comppsych.2015.07.004

[49] Stickley A, Koyanagi A, Takahashi H, Kamio Y. ADHD symptoms and pain among adults in England. Psychiatry Res. 2016;246: 326–331. 10.1016/j.psychres.2016.10.004 27750114

[50] Peles E, Schreiber S, Gordon J, Adelson M. Significantly higher methadone dose for methadone maintenance treatment (MMT) patients with chronic pain. Pain. 2005;113: 340–346. 10.1016/j.pain.2004.11.011 15661442

[51] Caldeiro RM, Malte CA, Calsyn DA, Baer JS, Nichol P, Kivlahan DR, et al The association of persistent pain with out-patient addiction treatment outcomes and service utilization. Addict Abingdon Engl. 2008;103: 1996–2005. 10.1111/j.1360-0443.2008.02358.x 18855809

[52] Sheu R, Lussier D, Rosenblum A, Fong C, Portenoy J, Joseph H, et al Prevalence and characteristics of chronic pain in patients admitted to an outpatient drug and alcohol treatment program. Pain Med Malden Mass. 2008;9: 911–917. 10.1111/j.1526-4637.2008.00420.x 18346064

[53] Nielsen S, Larance B, Lintzeris N, Black E, Bruno R, Murnion B, et al Correlates of pain in an in-treatment sample of opioid-dependent people. Drug Alcohol Rev. 2013;32: 489–494. 10.1111/dar.12041 23594352

[54] Barry DT, Beitel M, Joshi D, Schottenfeld RS. Pain and substance-related pain-reduction behaviors among opioid dependent individuals seeking methadone maintenance treatment. Am J Addict Am Acad Psychiatr Alcohol Addict. 2009;18: 117–121. 10.1080/10550490902772470 19283562 PMC2679697

[55] Darke S. Self-report among injecting drug users: a review. Drug Alcohol Depend. 1998;51: 253–263. 9787998 10.1016/s0376-8716(98)00028-3

